# Mutations in the Parkinson’s Disease-Associated PARK2 Gene Are Accompanied by Imbalance in Programmed Cell Death Systems

**Published:** 2015

**Authors:** E. V. Konovalova, O. M. Lopacheva, I. A. Grivennikov, O. S. Lebedeva, E. B. Dashinimaev, L. G. Khaspekov, E. Yu. Fedotova, S. N. Illarioshkin

**Affiliations:** Research Center of Neurology, Volokolamskoe Shosse, 80, Moscow, 125367, Russia; International Biotechnological Center, Lomonosov Moscow State University, Leninskie Gory, 1 /12, Moscow, 119991, Russia; Institute of Molecular Genetics, Russian Academy of Sciences, Kurchatov Square, 2, Moscow, 123182 , Russia; Koltsov Institute of Developmental Biology, Russian Academy of Sciences, Vavilova Str., 26, Moscow, 119334, Russia

**Keywords:** Parkinson’s disease, dopaminergic neurons, induced pluripotent stem cells, PARK2, mutation, programmed cell death

## Abstract

Parkinson’s disease is caused by the degeneration of midbrain dopaminergic neurons. A rare recessive form of the disease may be caused by a mutation in the *PARK2 *gene, whose product, Parkin, controls mitophagy and programmed cell death. The level of pro- and anti-apoptotic factors of the Bcl-2 family was determined in dopaminergic neurons derived from the induced pluripotent stem cells of a healthy donor and a Parkinson’s disease patient bearing *PARK2 *mutations. Western blotting was used to study the ratios of Bax, Bak, Bcl-2, Bcl-XL, and Bcl-W proteins. The pro-apoptotic Bak protein level in *PARK2*-neurons was shown to be two times lower than that in healthy cells. In contrast, the expression of the anti-apoptotic factors Bcl-XL, Bcl-W, and Bcl-2 was statistically significantly higher in the mutant cells compared to healthy dopaminergic neurons. These results indicate that *PARK2 *mutations are accompanied by an imbalance in programmed cell death systems in which non-apoptotic molecular mechanisms play the leading role.

## DISCUSSION

Parkinson’s disease (PD) is a common neurodegenerative disorder caused by lesions to pigmented neurons in the midbrain substantianigracompacta and degeneration of the dopaminergic nigrostriatal pathway. Treatment of PD is symptomatic and does not prevent further loss of nigral cells, which necessitates identification of the key elements in the disease pathogenetic cascade [1].

Genetics plays an important role in the development of PD. Monogenic forms account for approximately 10% of all PD cases [1, 2], while the other (sporadic) cases are of multifactorial nature. A total of 20 genetic PD loci have been identified to date [2]. One of the involved genes, *PARK2*, is located on the 6q25.2-27 chromosome and associated with the development of a particular form of the disease: an autosomal recessive PD, which is characterized by an early onset [3]. Mutations in *PARK2 *cause about 15% of familial and 4% of sporadic PD cases with the onset before the age of 40 years [3-5]. The *PARK2 *gene encodes cytosolic ubiquitin-E3- ligase, the Parkin protein. The main Parkin function is to regulate mitophagy. It acts in tandem with the PINK1 mitochondrial protein, which is a product of another gene of autosomal recessive Parkinson’s disease [6]. The sequence of molecular events occurs as follows: the dysfunctional mitochondrion is depolarized, thereby stabilizing PINK1; the latter recruits Parkin from the cytosol and activates it during its delivery to the mitochondrion, using the PINK1-kinase activity; then, the activated Park initiates selective autophagy of the damaged organelle [7].

Therefore, the interaction between Parkin and PINK1 controls the state of mitochondria. Currently, Parkin is regarded as a polyvalent neuroprotective agent that plays the key role in the survival of dopaminergic neurons upon exposure to various neurotoxins [8].

Studying *PARK2*/Parkin cell biology and neurochemistry is an extremely important area of modern neuroscience. The subject of our research is mutant Parkin expressed in cells of a patient with a rare* PARK2*-associated PD form. The dopaminergic neuron- enriched culture of nerve cells produced by reprogramming of dermal fibroblasts into induced pluripotent stem cells (iPSCs), followed by their directed differentiation, was used as a model system [9, 10]. In order to elucidate the disease pathogenesis, we examined the dynamics of several pro- and anti-apoptotic factors in the mutant cell culture in comparison to the neuron culture derived from a healthy donor.

We used cell cultures of a healthy donor and a patient with autosomal recessive juvenile-onset PD, who carried compound heterozygous mutations in the *PARK2 *gene (del202-203AG and IVS1+1G/A). Previously,we derived fibroblasts from skin biopsies of the donor and the patient. To generate iPCS clones, the fibroblasts were re-programmed using original lentiviral vectors with pluripotency factors (LeGO-hOCT4, LeGO-hSOX2-IRES-GFP, LeGO-hc-Myc, and LeGOhKLF4). The cells were then differentiated into the neuronal type using the differentiation factors. The techniques for producing and re-programming fibroblasts, as well as inducting differentiation of iPSCs into specialized dopaminergic neurons, were described previously [10].

Differentiation of cells into neurons was confirmed by immunohistochemical staining with antibodies to β-III-tubulin and tyrosine hydroxylase (TH). Western blotting, which was performed according to standard procedures, was used to determine the protein levels in cell cultures.

The results were processed using Microsoft Excel and the GraphPad Prism 6 software.

**Fig. 1:**
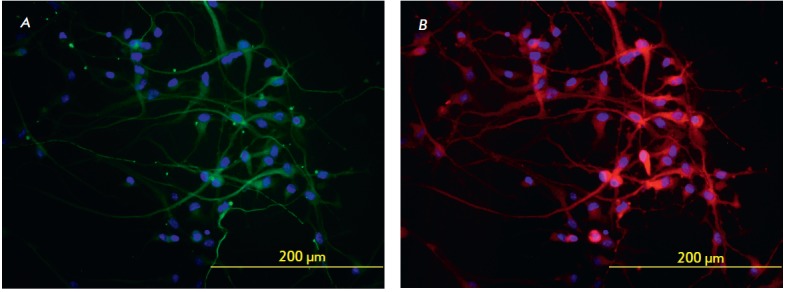
Immunohistochemical staining of iPSC-differentiated neurons. *A *–staining for β-III-tubulin (green); *B *– staining for TH (red); cell nuclei are shown in blue (DAPI staining)

During differentiation, Po2 and Tr5 cell lines expressed neuronal markers that were detected by immunohistochemical staining (*Fig. 1*). The β-III-tubulin protein is a classic marker of early neuronal differentiation, and TH is traditionally considered to be a specific marker of neuronal dopaminergic differentiation. As can be seen from *Fig. 1*, all iPSCs undergo differentiation since, all DAPI-stained cell nuclei are located within neurons (β-III-tubulin and TH-positive). The number of dopaminergic-differentiated neurons is approximately 80% of the total number of β-III-tubulinpositive cells.

**Fig. 2:**
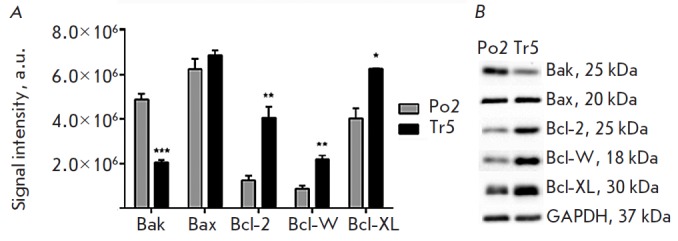
Quantitative analysis of pro- and anti-apoptotic proteins in the Po2 cell culture derived from a healthy donor and in the Tr5 culture derived from a PD patient carrying* PARK2 *mutations (Western blotting).* A *– signal intensity (arbitrary units) of Bak, Bax, Bcl-2, Bcl-W, and Bcl-XL bands (*n *= 3).*p * < 0.001 (***),* p * < 0.01 (**), and *p * < 0.05 (*) for comparison of Po2 and Tr5 cultures.* B *– representative pictures of Western blotbands

The ratio of pro-and anti-apoptotic factors is different in Po2 and Tr5 cell cultures derived from the healthy donor and the carrier of *PARK2 *mutations, respectively (*Fig. 2*).

The pro-apoptotic Bak protein level in Tr5 mutant dopaminergic neurons was statistically significantly lower (by 58%) than that in healthy donor cells in the Po2 culture (p < 0.001). No significant differences in the pro-apoptotic Bax protein level were observed in the cell cultures under study.

The levels of the anti-apoptotic proteins Bcl-2, Bcl- W, and Bcl-XL were statistically significantly higher in Tr5 cells expressing mutant *PARK2*. For example, the levels of the Bcl-2 protein, Bcl-W protein, and Bcl-XL protein in mutant cells compared to those in wild-type cells were 222% (p < 0.01), 150.5% (p < 0.01), and 55% (p < 0.05) higher, respectively.

Each sample contained 20 μg of the total protein. Uniformity of protein loading onto gel was additionally monitored using the GAPDH protein, whose signal level was similar in all samples (*Fig. 2B*).

Several variants of programmed cell death have been described for PD and other neurodegenerative diseases: classical apoptosis, autophagic pathway, AIF/ PARP-dependent pathway, paraptosis, etc [11, 12]. In some cases, however, cell death patterns do not correspond to a single mechanism and have a complex nature. The release of cytochrome *c *from mitochondria plays an important role in cell death [13]. Cytochrome* c *activates the cytosolic protein Apaf-1 and pro-caspase- 9 to form the apoptosome, and its release is strictly regulated in the cell by the pro- and anti-apoptotic factor ratio. The imbalance in the system, which we found in *PARK2*-compromised neurons, may be associated with the functional properties of defective Parkin, but this hypothesis needs further confirmation in cultures with normal and mutant genotypes.

The Parkin protein acts as a mitochondrial neuroprotector. In particular, a specific autonomic influence of Parkin on the mitochondrial mechanisms governing cytochrome *c *release and triggering apoptosis reactions was found [14]. Recently, a Parkin-like cytoplasmic protein associated with p53 (PARC) was identified; like Parkin, PARC is a E3-ligase and initiates proteasomal degradation of cytochrome *c *[15]. Proteasomal degradation of ARTS, which is a mitochondrial protein initiating an early (prior to the release of cytochrome* c*) step of the caspase pathway, may be an important stage in the protective action of Parkin [16]. However, the detailed molecular mechanisms of Parkin-associated neuroprotection still remain unclear.

A feature of the Tr5 culture is the lack of normal Parkin, since the neurons of this culture have two recessive inactivating mutations in the *PARK2 *gene (frameshift deletion del202-203A and IVS1+1G/A splicing mutation). Thus, the Parkin-mediated selective processing of mitochondrial proteins and cytochrome* c *is disrupted in the mutant culture. In this case, in the Tr5 culture, we found a statistically significant decrease in the pro-apoptotic Bak protein concentration instead of the expected initiation of apoptosis and, conversely, an increase in the level of all tested anti-apoptotic proteins of the Bcl family. These results are similar to the data on an increased Bcl-XL level *in vivo *and *in vitro*, in a paraquat-induced Parkinsonism model [17]. Prevention of apoptotic Bax protein translocation to the mitochondria is considered to be one of the functions of Parkin [18]; however, dysregulation of this process in *PARK2*-mutant cells does not affect the expression of Bax and may be not associated with the changes in its level in the culture. In recent years, microglial activation and inflammation have been considered to be important in the cytotoxic mitochondrial cascade in PD [19]; these processes do not require the induction of mitochondrial apoptosis [20], which is indirectly confirmed by our data.

## CONCLUSIONS

1. We characterized the Bax/Bak/Bcl system in the case of autosomal recessive PD with a complex *PARK2* mutation: *PARK2 *mutations were found to lead to a complex imbalance in programmed cell death systems, in which the non-apoptotic molecular mechanisms likely play the leading role.

2. These preliminary data need to be confirmed in dopaminergic neurons derived from other homozygous and heterozygous carriers of *PARK2 *mutations.

3. These findings demonstrate that approaches to the treatment of neurodegenerative diseases using inhibition of apoptosis [11, 12] may be ineffective in the case of *PARK2*-associated PD.
